# Collaborative Practices in Unnamed Housework and Childcare Among Dual-Earner Families: A Qualitative Descriptive Study on Family-to-Work Enrichment

**DOI:** 10.7759/cureus.92439

**Published:** 2025-09-16

**Authors:** Miyuki Ishii, Junko Honda, Yuko Shimoda

**Affiliations:** 1 College of Nursing Art and Science, University of Hyogo, Akashi, JPN; 2 Research Institute of Nursing Care for People and Community, University of Hyogo, Akashi, JPN; 3 Faculty of Nursing, Kyoto Tachibana University, Kyoto, JPN

**Keywords:** collaborative practices and implications, dual-earner families, family-to-work enrichment, invisible labor, public health nursing

## Abstract

Background: The growing prevalence of dual-earner households in Japan has attracted renewed attention due to the unequal distribution of domestic labor. In particular, many essential daily responsibilities, such as anticipating children's needs and managing household logistics, remain invisible and are cognitively challenging. These "unnamed" forms of housework and childcare are often overlooked in family health support practices despite their significant impact on caregiver well-being and work-family balance. For public health and family nursing professionals, addressing these hidden burdens is critical to promote sustainable family functioning.

Objectives: We aimed to explore how dual-earner couples with preschool-aged children comprehend, negotiate, and distribute invisible domestic labor and how these collaborative practices may promote family-to-work enrichment (F-to-WE). Specifically, this study aimed to (1) examine how dual-earner couples allocate and experience unnamed housework, (2) investigate how they allocate and experience unnamed childcare responsibilities, (3) identify collaboration patterns related to these tasks, and (4) analyze how these collaborative practices influence the psychosocial dynamics of work-family enrichment (WFE).

Methods: This qualitative descriptive study was conducted using semi-structured interviews with 15 dual-earner couples (30 participants) rearing preschool-aged children in Japan. Data were collected between April and June 2025 and analyzed using thematic coding to identify patterns of task sharing, emotional experiences, and perceived effects on work performance.

Results: The participants described unnamed domestic labor as emotionally and cognitively intensive. Five task-sharing typologies have emerged as follows: fixed division, complementary roles, flexible/mutual arrangements, visualization-based consensus, and crisis-driven restructuring. Couples who engaged in reciprocal support and spontaneous collaboration reported reduced mental load, improved emotional readiness, and enhanced engagement at work, which are the key characteristics of F-to-WE. In contrast, a lack of visibility or unequal task distribution leads to emotional exhaustion and strained relationships.

Conclusions: Invisible domestic labor significantly influences both family dynamics and occupational well-being. Public health nurses (PHNs) and family nursing practitioners can play pivotal roles in supporting couples in recognizing, redistributing, and jointly managing these tasks. Interventions such as task visualization tools and structured dialogue may enhance intra-family communication, reduce mental load, and promote mutually reinforcing work-family integration.

## Introduction

Dual-earner households have become increasingly prevalent in Japan in recent decades. According to data published by Japan's Ministry of Internal Affairs and Communications Statistics Bureau [1], the number of dual-earner households in 2024 was anticipated to increase by 220,000 from the previous year, reaching approximately 13 million. Since 1980, this figure has more than doubled as approximately six million dual-earner households were recorded. Despite this trend, the division of household labor remains imbalanced, with women shouldering housework and childcare. According to the 2021 Social Life Survey [2], wives in dual-earner households spend an average of seven hours and 28 minutes per day on these tasks, while their husbands spend only one hour and 54 minutes. Such disparities, compounded by perceptual discrepancies between spouses, may hinder effective collaboration and lead to dissatisfaction within households.

Against this backdrop, scholarly attention in Japan has increasingly focused on the concepts of "unnamed housework" and "unnamed childcare." These terms refer to small yet indispensable daily tasks such as anticipating children's needs, managing supplies, and coordinating transitions that are not typically categorized as formal household labor, such as cooking or cleaning [3]. Although each task may seem trivial, its cumulative nature and frequency make it emotionally and cognitively demanding [4]. These tasks are often invisible to non-performing partners; therefore, a lack of recognition can generate emotional strain, perceived inequity, and relational tension.

Although related concepts such as invisible labor, mental load, and emotional labor have been extensively discussed in Western literature, few studies have clearly defined and qualitatively examined "unnamed" housework and childcare, particularly from both partners' perspectives in dual-earner families [5,6]. Therefore, elucidating how couples recognize, allocate, and engage in these often-overlooked responsibilities in their everyday lives is essential. This focus is particularly important for parents with preschool-aged children, as this developmental stage is characterized by intensive childcare demands, frequent transitions in daily routines, and heightened negotiation of household responsibilities. These circumstances often exacerbate invisible labor, disproportionately affecting mothers and creating challenges for equitable collaboration.

To address these gaps, this study uses the work-family enrichment (WFE) theoretical framework, which describes how experiences in one area (such as family) can improve performance in another (such as work) [7]. WFE is conceptualized as bidirectional, consisting of family-to-work enrichment (F-to-WE) and work-to-family enrichment (W-to-FE). Interpersonal support, emotional communication, and sharing psychological resources within families can positively influence individuals' workplace well-being and performance [8]. However, existing studies have primarily focused on formal role arrangements or general family support, with limited exploration of how the microlevel management of invisible domestic labor contributes to enrichment processes.

In the context of Japanese community health, public health nurses (PHNs) play a pivotal role in promoting family well-being by providing health checkups, conducting home visits, offering parenting education, and implementing community-based programs. Recently, there has been a paradigm shift in nursing and public health practice from managing work-family conflict to fostering positive interactions between work and family domains, as defined by the WFE concept [9,10].

Understanding the dynamics of "unnamed" domestic labor is critical for developing effective, family-centered nursing interventions. We examined how dual-earner couples perceive, negotiate, and manage invisible tasks. These findings may contribute new knowledge in the fields of family and public health nursing and occupational health. They also offer practical insights for PHNs seeking to promote equitable domestic collaboration and mutually reinforce work-family dynamics.

Therefore, this study aimed to explore how dual-earner couples with preschool-aged children perceive and collaboratively manage "unnamed housework" and "unnamed childcare" in their daily lives, and how these practices contribute to the formation of WFE, especially in the family-to-work direction (F-to-WE). Specifically, this study aimed to (1) examine how dual-earner couples allocate and experience unnamed housework, (2) investigate how they allocate and experience unnamed childcare responsibilities, (3) identify collaboration patterns related to these tasks, and (4) analyze how these collaborative practices influence the psychosocial dynamics of WFE.

## Materials and methods

Operational definition of key terms

In this study, specific terms were operationally defined, as shown in Table 1. For clarity in reporting, "unnamed housework" generally includes small, routine, and often invisible tasks such as tidying up, managing household supplies, or making minor household adjustments. Examples were not presented to the participants, as the aim of the study was to elicit their perceptions of "unnamed" housework.

**Table 1:** Operational definition of key terms

Term	Operational definition
Unnamed housework	Routine household tasks that are frequently overlooked or taken for granted
Unnamed childcare	Routine childcare tasks that are frequently overlooked or taken for granted
Housework and childcare collaboration	Shared responsibilities associated with household tasks and childcare
Dual-earner couples	Couples in which both partners were engaged in paid employment

Study design

A qualitative descriptive study design was employed because it is suitable for exploring relatively new research areas [11]. This approach allows researchers to describe participants' experiences and everyday practices in simple language, making the findings easier to understand and apply. This design was considered particularly appropriate for examining the recognition and sharing of "unnamed" housework and childcare, which have not been extensively studied in the context of dual-earner families in Japan.

Participant recruitment

The study participants were couples who satisfied the following criteria: (1) both individuals were responsible for raising at least one child of preschool age, which is defined as zero to six years of age in Japan; (2) both individuals were employed for an average of at least seven hours per day, five or more days per week; (3) the participants, along with their spouses/partners and children, cohabitated; and (4) both participants and their spouses/partners consented to participate in the interview. Marital status and work style (e.g., office-based or remote work) were not used as exclusion criteria.

Participants were recruited using a purposive sampling strategy that used the researchers' professional and personal networks. Invitations to participate were disseminated through the administrative offices of a public health nursing seminar and a Master of Business Administration alumni reunion. Potential participants received an informational leaflet and study overview through email. These materials comprehensively outlined the study's purpose, eligibility criteria, procedures, contact information, and the voluntary nature of participation. The leaflet explicitly clarified that the study was independent of the seminar or the reunion and that non-participation would not impact seminar or reunion attendance or associated benefits. Interested individuals were instructed to contact the researchers directly. Fifteen couples (30 individuals) who satisfied all the inclusion criteria voluntarily consented to participate in the study.

Sample size and data saturation

In line with the qualitative descriptive methodology, a purposive sampling strategy was employed to recruit couples with diverse occupations and family structures. A total of 15 couples (30 individuals) were recruited, which was considered sufficient to capture variations in experiences while remaining manageable for in-depth analysis. The adequacy of the sample was confirmed through the principle of data saturation: recurring themes and no emergence of substantially new insights were observed before completing 30 interviews. This supports the appropriateness of the sample size for this study.

Development of the interview guide

A semi-structured interview guide and draft questions were developed and refined through discussions among the research team to ensure the clarity and relevance of the content. This process ensured that the questions were appropriate for eliciting rich narratives, although they were not intended to function as psychometrically validated instruments, as in quantitative studies. The final version of the guide included six open-ended questions (Table 2).

**Table 2:** Semi-structured interview questions

Number	Question
1	Could you share any specific examples of tasks that you usually perceive as "unnamed" housework or "unnamed" childcare?
2	How do you and your spouse/partner typically share the responsibilities for those tasks?
3	Are there any aspects of how tasks are divided between you and your partner that you are particularly satisfied with, or any areas you feel could be improved?
4	In what ways do you and your partner endeavor to demonstrate consideration for one another when engaging in shared responsibilities related to household tasks and childcare?
5	What approaches or techniques do you and your partner use to effectively manage and collaborate on household and childcare duties?
6	Have you experienced any positive effects at work that stem from how household and childcare responsibilities are managed in your home?

Data collection

Interviews with the guide were conducted between April and June 2025 using an online conferencing platform (Zoom; Zoom Video Communications, San Jose, CA). Thirty participants (15 couples) were interviewed individually, with each session lasting 35-60 minutes. The number of participants was not predetermined; recruitment and analysis proceeded concurrently, and interviews continued until no new themes emerged and thematic saturation was achieved. At that point, the total number of participants was 30, which was considered sufficient for the study's aims and qualitative descriptive analysis. All interviews were recorded with the participants' consent prior to conducting the interview.

Data analyses

The interview data were transcribed verbatim and analyzed using a qualitative descriptive approach with open coding methods. Two researchers independently coded the transcripts line by line to identify meaningful units related to "unnamed housework," "unnamed childcare," and dual-earner couples' collaborative practices. The codes were grouped into subthemes based on semantic similarities and then integrated into broader themes through iterative comparisons of the data.

To ensure credibility, a third researcher reviewed the coding framework and the resulting themes. The three researchers held multiple discussions to compare interpretations, resolve discrepancies, and refine the definitions of subthemes and themes until a consensus was reached. This collaborative process minimized individual bias and strengthened the trustworthiness of the analyses.

Ethical consideration

This study was approved by the Research Ethics Committee of the University of Hyogo (approval number: 2024F19). All participants were informed of the study purpose and policies regarding the protection of their personal information before providing written informed consent.

## Results

The findings of this study are presented based on a qualitative descriptive analysis of the interview data. Rather than quantitative results or statistical tests, the outcomes were articulated through themes and typologies that emerged from participants' narratives. This approach reflects the aim of qualitative descriptive research, which is to provide a comprehensive and nuanced understanding of participants' experiences in their own words.

The Results section is organized to reflect the four study objectives stated in the Introduction section. Specifically, Attributes of the study participants and Recognition of unnamed housework and childcare correspond to objectives 1 and 2, Typology of unnamed housework and childcare division corresponds to objective 3, and Work-family enrichment through collaborative practice addresses objective 4.

Attributes of the study participants

The attributes of the study participants are listed in Table 3. The mean age values of the wives and husbands were 33.2 and 34.8 years, respectively. With respect to the number of children, 10 households had one child, three households had two children, and two households had three children. All households were nuclear families. The occupations of the wives included company employees, nurses, PHNs, childcare workers, teachers, and university faculty members. The occupations of the husbands included company employees, self-employed workers, teachers, public officers, researchers, and university faculty members. The participants were residents of Tokyo, Osaka, Kyoto, Kobe, or nearby municipalities.

**Table 3:** Attributes of the study participants 0: wife, 1: husband

ID	Pair	Age	Age and sex of children	Occupation	Work shift
A1	0	32	Male (2-years-old)	Nurse	Day
A2	1	30		Self-employed worker	Day
B1	0	32	Female (1-year-old), male (2-months-old)	Company worker	Day
B2	1	34		Company worker	Day
C1	0	30	Female (2-years-old)	Nurse	Day
C2	1	33		Company worker	Day
D1	0	37	Female (7-years-old), male (4-years-old)	Junior high school teacher	Day
D2	1	35		Junior high school teacher	Day
E1	0	28	Male (1-year-old)	Childcare worker	Day
E2	1	28		Public office worker	Day
F1	0	39	Male (4-years-old)	University faculty member	Day
F2	1	44		University faculty member	Day
G1	0	31	Male (6-years-old), female (3-years-old), female (1-year-old)	Company worker	Day
G2	1	32		Company worker	Day
K1	0	28	Male (1-year-old)	Public health nurse	Day
K2	1	29		Company worker	Day
L1	0	30	Female (2-years-old)	Nurse	Day and night
L2	1	33		Nurse	Day and night
M1	0	35	Male (3-years-old)	Company worker	Day
M2	1	35		Company worker	Day
N1	0	43	Male (9-years-old), female (4-years-old)	Company worker	Day
N2	1	48		Researcher	Day
O1	0	31	Female (1-year-old)	Company worker	Day
O2	1	33		Company worker	Day
R1	0	29	Female (3-years-old)	Nurse	Day
R2	1	30		Elementary school teacher	Day
S1	0	40	Female (12-years-old), male (9-years-old), male (4-years-old)	Company worker	Day
S2	1	42		Company worker	Day
T1	0	34	Female (1-year-old)	Company worker	Day
T2	1	36		University faculty member	Day

Recognition of unnamed housework and childcare

The participants identified "unnamed housework" and "unnamed childcare" not merely as physical tasks, but rather as multifaceted practices involving emotional and cognitive efforts. These acts were challenging for those performing them to visualize or articulate because of their internalization as a component of everyday life.

Based on a comprehensive analysis of narratives of the participants, four major themes were identified regarding how such tasks are perceived and understood as follows: (1) tasks that are "invisible" or "easily overlooked," (2) tasks perceived by the performer as routine and meaningful, (3) differences in awareness between spouses, and (4) emotional and cognitive labor. The following sections describe each theme in detail. Table 4 shows each theme and representative quotes.

**Table 4:** Recognition of unnamed housework and childcare

Themes	Representative quotes
Tasks that are "invisible" or "easily overlooked"	D1: *"Taking out the trash, getting the kid to daycare, tidying up afterwards... I think those are examples of unnamed housework and childcare."*
	T1:* "For example, I separate the clothes for laundry, I know which set needs to be done by a certain day, or I remember which underwear will run out by a certain day, so I need to do a load by then. I also remember which items can or can't go in the dryer. I guess that's what you'd call unnamed housework."*
	A1: *"I feel like it's just assumed I'll do it. It's like when I'm doing daycare paperwork or writing in the communication notebook: it's just what I do, and I never get thanked for it."*
Tasks perceived by the performer as routine and meaningful	G2:* "They call it 'unnamed housework,' but to me, it's clearly defined. It's not that it doesn't have a name: it's just that others don't see it. For me, it's part of my routine, and it has real meaning."*
	S2: *"When I hear 'unnamed,' it sounds like something that's being overlooked, but to me, it's part of my work. It's not so much that it lacks a name, more like it's something that needs explaining for others to understand."*
Differences in awareness between spouses	T2: *"My wife said, 'My husband doesn't even know what unnamed housework is.' To be honest, I used to only think about the 'visible' stuff, you know, like cooking and laundry. But lately, I've been realizing, 'Oh, that was housework too.'"*
	M2:* "I'd just assume they were already prepared when I checked the daycare items and such. But once I actually had to do it myself, I realized, 'Wow, this takes a lot more effort than I thought.'"*
Emotional and cognitive labor	G2: *"Before my kid starts saying 'no,' I try to change how I say things to gently guide them. I also try not to disrupt the morning flow with how I speak. That kind of thing probably takes the most mental energy."*
	C1: *"While I'm watching to see if they're going to eat, I try putting the spoon back in their mouth or offering it again at the right moment. I feel like this kind of adjusting to their mood is the most 'parenting-like' part of childcare."*

Tasks That are "Invisible" or "Easily Overlooked"

Many participants identified tasks such as sorting refuse, separating laundry, checking daycare items, and writing in communication notebooks as forms of "unnamed housework" and "unnamed childcare" because they are often unseen and easily overlooked. These tasks are typically executed within the domestic sphere. Consequently, their significance is frequently overlooked and underappreciated by other family members.

Tasks Perceived by the Performer as Routine and Meaningful

Some participants expressed that they weren't comfortable with the term "unnamed," and they emphasized that these tasks are clearly and meaningfully part of their daily routines. They said that the problem was not that these tasks did not have names, but that they were invisible to others. Their comments indicated that they were dealing with internal conflicts. On one hand, they said that these actions were meaningful to them, but on the other hand, they often went unrecognized by their partners.

Differences in Awareness Between Spouses

Participants described discrepancies in awareness among spouses concerning "unnamed housework" and "unnamed childcare." A significant number of wives reported engaging in these tasks unconsciously and noted that their husbands either did not notice or did not consider them as housework. Concurrently, several husbands disclosed that they had only recently become cognizant of the actions of their spouses. This realization frequently emerged after the first execution of these tasks, which invariably exposed the immense effort required.

Emotional and Cognitive Labor

"Unnamed childcare" was perceived as more than just a physical task, such as writing in communication notebooks. It was also perceived as an emotionally and cognitively demanding activity, such as adjusting one's tone to match the child's mood or preparing in ways that make it easier to have a smooth morning routine. These acts are comprehended as the key components of caregiving.

Typology of unnamed housework and childcare division

A comprehensive analysis of narratives of the participants led to the identification of five distinct typologies of task-sharing structures (Table 5). These typologies are determined by the initiating factors of division, involvement degree, and flexibility of roles.

**Table 5:** Typology of unnamed housework and childcare division

Typologies	Representative quotes
Fixed division type	A1: *"I'm the one handling everything, submitting daycare forms, keeping up with the communication notebook, so it's no surprise that no one ever says 'thank you.'"*
Complementary division type	G2: *"I try to pitch in where I can, like taking out the trash or cleaning the bathroom."*
Flexible and mutually complementary type	N1: *"We coordinate based on our daily rhythms and skills, like whoever wakes up earlier takes care of the trash, my wife handles the cooking since she's good at it, and I take care of the cleanup."*
Visualization and consensus-building type	E1: *"Before I went back to work, my husband and I made a list of all the things we needed to do around the house and put it on the fridge."*
Reconstructed type	M1:* "I was totally overwhelmed and couldn't take it anymore, so I said, 'Please help,' and that's when my husband started hanging the laundry."*

Fixed Division Type

In this type of marriage, the majority of domestic responsibilities, including housework and childcare, are shouldered by the wife, while the husband's involvement is characterized by irregularities or limitations. Daily roles are typically established through unilateral decision-making and seldom undergo reevaluation.

Complementary Division Type

In this household, the primary responsibility for domestic work and childcare falls on the wife, while the husband contributes to the household in a supplementary or voluntary capacity. A common phenomenon is the tendency of husbands to assume routine or physically demanding tasks, thereby attempting to redress the perceived imbalance in familial responsibilities.

Flexible and Mutually Complementary Type

The distribution of tasks is characterized by flexibility, with adjustments to the schedule, strengths, and preferences of each spouse. The absence of formal division charts and explicit rules is notable. Instead, mutual support is practiced in an organic and adaptive manner.

Visualization and Consensus-Building Type

This approach entails the systematic documentation of all domestic and childcare responsibilities, either through list creation or chart utilization. The information is subsequently shared by the couple, facilitating the clarification of responsibilities and establishment of a collaborative foundation.

Reconstructed Type

This typology captures cases in which task sharing initially followed a rigid, wife-centered division but underwent a notable transformation. This shift is often prompted by psychological or physical limitations of the wife, eventually resulting in a more balanced and flexible arrangement. While current practices may resemble the flexible and mutually complementary type, the transition process itself is noteworthy and distinguishes this type.

Work-family enrichment through collaborative practice

Beyond the categorization of perceptions and task-sharing structures, the present analysis also examined how collaborative involvement of the participants in "unnamed" housework and childcare contributed to their experiences of WFE, particularly in the F-to-WE direction.

Several participants articulated that equitable and mutually supportive domestic arrangements mitigated their emotional distress, streamlined their morning routines, and augmented their psychological readiness for the workday. Despite the informal and organic nature of these collaborative practices, they were perceived as generating a positive spillover from the family domain to the work domain.

## Discussion

The present study examined how dual-earner couples with preschool-aged children identify, distribute, and collaboratively manage "unnamed" domestic tasks and childcare, and how these practices relate to WFE. A qualitative descriptive analysis of 15 couples yielded the identification of five typologies of task-sharing structures. Furthermore, analysis of the four major themes of recognition yielded insights into the emotional and cognitive dimensions of invisible labor. These findings elucidate how quotidian, frequently disregarded as collaborative practices within the domestic sphere, can engender psychological well-being and relational equilibrium with auspicious ramifications for occupational functioning, particularly in the F-to-WE direction.

Invisible labor as cognitive and emotional work

The participants concurred that housework and childcare are not merely physical tasks but also involve substantial emotional and cognitive labor. Examples of these behaviors include anticipating a child's needs, organizing daycare materials, and adjusting to the child's tone to prevent conflict. These actions, although often executed unconsciously, remain unacknowledged by partners. This discrepancy in awareness between those who perform tasks and those who benefit from them frequently contributes to emotional fatigue and a sense of underappreciation, particularly among female partners. These findings are consistent with prior research on "mental load" and "invisible labor" [12], which delineate how gendered expectations and lack of visibility perpetuate inequities in domestic labor. In the Japanese context, although dual-earner households have become increasingly common, traditional gender norms continue to shape the division of domestic and childcare responsibilities, with women frequently expected to assume the primary caregiving role. Furthermore, while childcare services have expanded in recent years, their availability and accessibility remain uneven across regions, which may exacerbate the invisible labor disproportionately borne by mothers.

Task-sharing structures as dynamic and context-driven

The five typologies identified (fixed division, complementary division, flexible mutual support, reconstructed division, and visualization-based consensus) demonstrate that household task allocation is shaped by both structural and interpersonal factors. The reconstructed division materialized not as a consequence of deliberate negotiation but rather as a result of pivotal junctures such as burnout or health constraints. Conversely, visualization-based consensus exhibits a proactive and communicative approach.

These observations suggest that task sharing is not a static agreement, but a dynamic, evolving process often shaped by emotional feedback, life events, and mutual adjustment. This finding aligns with the conclusions of Dunn and O’Brien, who posited that gendered task division undergoes adaptive changes over time in response to life transitions [13]. Additionally, it resonates with Hochschild and Machung's seminal work on the "economy of gratitude," which underscores the pivotal role of mutual recognition in fostering cooperation [14].

According to this study findings, couples classified under the flexible and mutually complementary themes exhibited enhanced coordination and emotional equilibrium, which are recognized as the psychological underpinnings of WFE. These findings underscore the notion that adaptability and continuous renegotiation of domestic roles can mitigate role strain, thereby facilitating enhanced integration between family and work life.

Collaborative practices and family-to-work enrichment

These findings also substantiate the theoretical proposition that collaborative domestic practices can generate positive spillover effects in the workplace. The participants articulated that the distribution of shared responsibility and provision of emotional support within the domestic environment augmented their psychological preparedness for the workday, thereby leading to a reduction in their overall stress levels. These collaborative practices, characterized by their informal and emergent nature, foster relational equity and emotional availability, thereby contributing to WFE [7].

Notably, intentional collaboration and mutual recognition have emerged as critical psychosocial mechanisms by which domestic cooperation enhances occupational functioning. Couples who engage in deliberate efforts to distribute responsibilities or express appreciation, whether through the implementation of checklists or the introduction of spontaneous routines, exhibit increased confidence, diminished role strain, and enhanced emotional resilience. This finding aligns with the conclusions of Carlson et al., who underscored the pivotal role of family support in facilitating work engagement [8], and Chen et al., who demonstrated that spousal support enhances psychological well-being and job satisfaction [15].

Conversely, an asymmetrical or unrecognized task division was associated with emotional fatigue and diminished relational satisfaction. These findings suggest that practices that are visible, equitable, and mutually endorsed may be essential not only for achieving gender equality but also for supporting sustainable dual-earner dynamics and preventing work-family conflict.

These findings directly correspond to the study objectives. Specifically, the typologies of task-sharing structures address objectives 1 and 2 by illustrating how couples allocate and experience unnamed housework and childcare. Objective 3 is reflected in the identification of collaboration patterns, such as flexible mutual support and visualization-based consensus. Finally, objective 4 is addressed through the analysis of how these collaborative practices influence family-to-work enrichment, highlighting the psychosocial mechanisms that underpin WFE. By explicitly linking the objectives to the findings, this study demonstrates how the microlevel management of invisible domestic labor contributes to the broader theoretical framework of WFE.

Practical implications for public health and family support

The findings have significant implications for public health nursing and family interventions. PHNs are uniquely positioned to identify and address the emotional burden of invisible labor during home visits or parenting support programs. The promotion of discourse concerning "unnamed" responsibilities and the cultivation of mutual recognition have the potential to enhance familial functionality and relational satisfaction.

Furthermore, the typology developed in this study may inform the design of psychoeducational interventions, including household task visualization tools, collaborative planning workshops, and communication-based support sessions. By enhancing the awareness of couples and renegotiating household responsibilities, PHNs and family support professionals can help promote equitable collaboration and foster WFE.

Limitations and future research

This study had some limitations. First, the participants were relatively well-educated urban couples, which may limit the generalizability of the findings to more diverse socioeconomic and rural populations. Second, the use of individual interviews limited our ability to observe direct dyadic interactions and negotiation processes among spouses. Future research should consider dyadic or longitudinal methods, such as joint interviews or diary studies, to capture the evolving dynamics of task sharing more comprehensively.

Furthermore, quantitative approaches, including actor-partner interdependence models and structural equation modeling, may prove instrumental in elucidating the causal pathways through which collaborative practices contribute to WFE. A mixed-methods approach could provide a more nuanced understanding of how invisible labor contributes to psychological well-being and occupational functioning over time.

## Conclusions

This study highlights the cognitive and emotional dimensions of "unnamed" housework and childcare among dual-earner couples in Japan, as well as the collaborative practices that foster family-to-work enrichment (F-to-WE). The typology of task-sharing structures developed herein underscores that domestic labor is not static but evolves through interpersonal negotiation, life events, and mutual recognition. Our findings indicate that equitable and visible collaboration reduces stress, strengthens emotional balance, and enhances work engagement, thereby contributing to sustainable integration between family and work. These results suggest that nursing and public health interventions should address invisible domestic labor as a key determinant of family well-being. Public health nurses are uniquely positioned to promote awareness, encourage mutual recognition, and support family-centered strategies that enhance the F-to-WE.

## References

[1] (2025). Statistics Bureau, Ministry of Internal Affairs and Communications: Labor force (detailed statistics) survey. https://www.stat.go.jp/data/roudou/sokuhou/nen/dt/pdf/gaiyou.pdf.

[2] (2025). Ministry of Internal Affairs and Communications: Basic survey on social life. https://www.stat.go.jp/data/shakai/2021/pdf/youyakua.pdf.

[3] Takayama J (2020). Difficulties and challenges of dual-earner couples in sharing and managing housework during the child-rearing period (Article in Japanese). Ann Fam Stud.

[4] Shimazu A, Fujiwara T, Iwata N (2023). Effects of work-family life support program on the work-family interface and mental health among Japanese dual-earner couples with a preschool child: a randomized controlled trial. J Occup Health.

[5] Ciciolla L, Luthar SS (2019). Invisible household labor and ramifications for adjustment: mothers as captains of households. Sex Roles.

[6] Rissing A, Inwood S, Stengel E (2021). The invisible labor and multidimensional impacts of negotiating childcare on farms. Agric Human Values.

[7] Greenhaus JH, Powell GN (2006). When work and family are allies: a theory of work-family enrichment. Acad Manag Rev.

[8] Carlson DS, Kacmar KM, Wayne JH, Grzywacz JG (2006). Measuring the positive side of the work-family interface: development and validation of a work-family enrichment scale. J Vocat Behav.

[9] Kawakita T, Hosoda Y (2024). Work-family enrichment among parent nurses: a cross-sectional scale development and validation study. BMC Nurs.

[10] Shimoda Y, Ishii M, Hori Y (2023). Challenges in work-family balance and support needs of Japanese parents with nursery school-aged children. Health.

[11] Polit D, Beck C (2020). Nursing Research: Generating and Assessing Evidence for Nursing Practice. Nursing Research, 11th edition.

[12] Daminger A (2019). The cognitive dimension of household labor. Am Sociol Rev.

[13] Dunn MG, O'Brien KM (2013). Work-family enrichment among dual-earner couples: can work improve our family life?. J Couns Psychol.

[14] Hochschild A, Machung A (2012). The Second Shift: Working Families and the Revolution at Home.

[15] Chen Z, Powell GN, Greenhaus JH (2009). Work-to-family conflict, positive spillover, and boundary management: a person-environment fit approach. J Vocat Behav.

